# Effects of High-Temperature Storage on the Elasticity Modulus of an Epoxy Molding Compound

**DOI:** 10.3390/ma12040684

**Published:** 2019-02-25

**Authors:** Ruifeng Li, Daoguo Yang, Ping Zhang, Fanfan Niu, Miao Cai, Guoqi Zhang

**Affiliations:** 1 School of Automation, Beijing University of Posts and Telecommunications, No.10 Xitucheng Road, Beijing 100876, China; liruifeng8107@163.com; 2 School of Mechanical and Electrical Engineering, Guilin University of Electronic Technology, No. 1 Jinji Road, Guilin 541004, China; 1601201008@mails.guet.edu.cn (F.N.); caimiao105@163.com (M.C.); g.q.zhang@tue.nl (G.Z.)

**Keywords:** high-temperature storage, epoxy molding compound, elasticity modulus, microelectronics reliability

## Abstract

Changes in the elasticity modulus of an epoxy molding compound (EMC), an electronic packaging polymer, under high-temperature air storage conditions, are discussed in this study. The elasticity modulus of EMC had two different compositions (different filling contents) under different temperatures (175, 200, and 225 °C) and aging times (100, 500, and 1500 h), which were analyzed by using dynamic mechanic analysis technology. The results revealed that the elasticity modulus increased in the thermal aging process, with an increase in the temperature and the aging time. The increments of the glassy and rubbery states were similar. However, the growing rate was significantly different, and the growth of the rubbery state was significantly higher than that of the glassy state. The filling content influenced the degree of aging of the materials significantly. At a low filling content, long-term aging under high temperatures completely changed the material structure, and the mechanical properties of the polymer were reduced.

## 1. Introduction

Semiconductor packaging is used for most electronic equipment and product applications, including information terminals (cell phones and personal computers), digital cameras, video game consoles, and household electrical appliances (refrigerators, laundry machines), with an annual increase in the extent of its applications. Epoxy molding compounds (EMCs) for semiconductors are used for protecting semiconductor chips from the external environment, particularly physical forces, such as impact and pressure, and chemical forces, such as moisture, heat, and ultraviolet rays. This maintains the electrical insulation properties of the semiconductors, and provides a form for easy mounting of the semiconductor onto a printed circuit board [1].

The process of producing epoxy molding compounds for microelectronic packaging requires high-temperature transfer molding methods, followed by rapid cooling of the components to ambient temperature. This thermal cycle is repeated several times before the device is used. Additional heat treatments include post-mold curing at 175 °C for 4 h, solder dipping at 260 °C for 2 min, and vapor phase reflow methods. Post-mold cure cycles at temperatures that are close to the molding temperature over various times intervals are performed for varying time intervals. They are performed to optimize the properties of the molding materials [2]. These properties include *T*_g_, dielectric properties, insulation capability, and adhesion properties.

In addition, during operation, the microelectronic product can be exposed to temperatures that approach or are greater than the molding temperature. For example, replacing the existing hydraulic control brake with an electronically controlled brake is a future objective in the aerospace industry, with the aim of decreasing the weight, simplifying maintenance, and improving reliability. However, this requires the electronic elements to be adaptable to a high-temperature (>200 °C) environment [3]. The rapid development of the automobile industry in recent years has resulted in automobiles being equipped with electronic control units and intelligent sensors, to enhance control over the engine, the transmission, and the steering gear. To avoid electromagnetic compatibility, these electronic devices are generally installed in close proximity to the engine, and below the car hood, where the working temperature is approximately 150 °C [4,5]. The internal temperatures of the electronic devices are higher, generally being greater than 175 °C. Therefore, there is an increasing need for studies into the high-temperature aging of electronic devices.

High-temperature storage over a long duration could cause degradation of the epoxy resin used in molding compounds. This would result in significant changes in the mechanical properties of EMCs after oxidation. The presence of oxygen results in thermo-oxidative decomposition, and this accelerates failure in epoxy composite materials [6]. The diffusion of oxygen and its reaction with the polymer network results in the formation of an oxidized layer. The structural changes that occur in the oxidized layer induce material embrittlement on two levels. (1) On the macromolecular scale; chain scission and crosslinking alter the mechanical properties of the oxidized layer and particularly the fracture properties. A decrease in the break strain occurs. (2) On the macroscopic scale, the oxidized layer is denser, which causes shrinkage. The oxidized layer strongly adheres to the non-degraded core, and the shrinkage, combined with the altered mechanical properties, induces a tensile stress gradient that can result in the cracking of the oxidized layer [7].

Many studies have primarily focused on the experimental characterization of the thermal aging behavior of epoxy molding compounds, and their effects on the reliability of microelectronics. The effect of post-cure- and thermal aging on the mechanical properties of molding compounds has been reported in the literature [8,9]. The visco-elastic mechanical properties of two commercial EMC materials has been measured as a function of aging time, and the effect of EMC curing during its lifetime on the package reliability is reported [10]. Many researchers have investigated the effects of high-temperature aging on the thermo-mechanical properties and microstructures of the molding compounds, and finite element models have been established to simulate aging effects on the stress and strain of the packaging [11,12,13,14]. Cui developed a new numerical method for simulating the thermal oxidation–diffusion of epoxy molding compounds, based upon the similarities between oxidative diffusion and heat transfer equations [15]. An "equivalent layer" model, which includes a fully oxidized layer and an unaged core, was proposed by Bingbing. It was applied to simplify the modeling of thermal aging effects. The thickness of the "equivalent oxidized layer" is obtained by combining the experimental results and the numerical analyses of properly chosen samples. Additionally, the aging shrinkage is estimated by using the equivalent thickness concept [16].

Epoxy molding is a viscoelastic material, which possesses mechanical properties that include both the elasticity of a rigid body and the viscosity of a fluid, in the storage modulus, and in the loss modulus, respectively. The storage modulus (elasticity modulus) is an important performance parameter in engineering materials, and it directly influences the use and technological performance of electronic polymers. It is the parameter that is used to calculate material stress and strain. A study on the mechanical properties (particularly with regard to the changes in the elasticity modulus) of epoxy molding compounds under high-temperature storage requires the construction of a thermal oxidation model, and numerical simulations need to be performed to calculate the stress and strain of the material during thermal aging [12,14]. Then, the lifetime of the electronic components can be predicted.

However, only a few reports have been made on the elasticity modulus of EMCs under high-temperature storage. Only a few articles have demonstrated variations in the elasticity modulus with aging time under a certain temperature. In fact, the composition and the aging temperature also have great influences on the elasticity modulus. Therefore, a systematic study into the variation of the elasticity modulus with composition, temperature, and time is needed. In this study, the effects of temperature, time, and the filling contents of the elasticity modulus of the EMCs under high-temperature aging conditions were investigated. A qualitative analysis of the relationship between the thermal aging evolution of the elasticity modulus and material failure was performed.

## 2. Experiment

### 2.1. Sample Preparation

The main ingredients of EMCs are epoxy resin, fused silica as a filler, a hardener resin, a cure promoter, a coupling agent, a flame retardant, and other additives. Two types of EMC samples with different filling contents (wt.%) were chosen: material 1 had a high filler content of 89%, and material 2 had a low filler content of 79%. The filler used was spherical fused silica with a sieving size of 100 μm. The raw materials were mixed and kneaded under heating into a homogeneous mixture, in a kneader or a roll mixer. The materials were kneaded during cooling into a sheet, which was then pulverized. The powdery material was palletized into pellets, which were used in the transfer molding step.

A large number of samples were prepared for each material with the same composition and applied into the same experimental conditions to ensure the repeatability of the experimental results. All of the samples were made by using the transfer molding process, which was the same as that which was used in the industry to ensure the accuracy of the composition. The transfer molding process included: (a) a tight closure of the mold die, and heating the mold cavity; (b) feeding EMC pellets through the mold pot under pressure with a plunger into each cavity; (c) maintaining compression of the EMC in the cavity until it is cured; and (d) opening up the mold, releasing the molded package. The in-mold curing temperature was 175 °C, and the post mold cure incubation lasted for 4 h at 175 °C. The molded samples were bar-shaped, approximately 50 mm × 10 mm × 2 mm.

The high-temperature storage experiments of the materials were performed in air chambers at three temperatures and three aging times. The experimental conditions were as follows (Table 1):

### 2.2. Experimental Methods

A DMA(Dynamic Thermo-mechanical Analysis) was conducted, to characterize the temperature dependence of the materials. DMA2980 (TA Instrument Co., Ltd., Wilmington, Delaware, USA) was used in the single cantilever mode. DMA testing of all the non-aged and aged samples at 1 Hz (1/s) was performed. The temperature scanning ranged from 25 to 300 °C.

## 3. Results and Discussion

**Figure 1:**
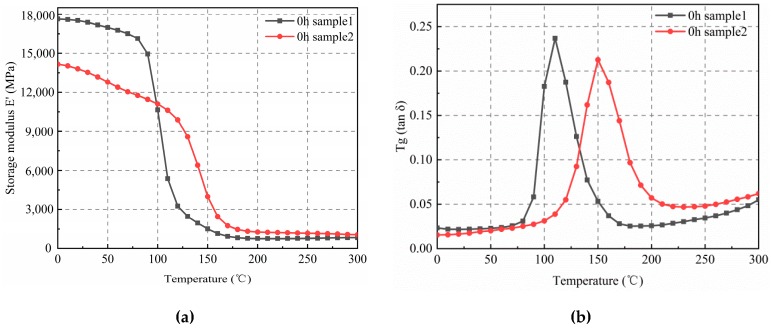
Variation curves of the elasticity modulus, and *T*_g_ of the non-aged (0 h) samples. (**a**) E’–T curves; (**b**) tan δ–T curves.

Figure 1 and Table 2 show that before aging (0 h), the mean modulus of sample 1 in the glassy state was higher than that of sample 2, and the mean modulus of sample 1 in the rubbery state was lower. This was related to the higher filling content of sample 1. EMC is a composite material that is composed mainly of epoxy resin and silica-filling materials. As the elasticity modulus of silica is significantly higher than that of epoxy resin, the material with the higher filling content had the higher modulus in the glassy state. Moreover, an excess of silica content affected material curing, decreasing the *T*_g_ value of the composite material (Table 2). The elasticity modulus of sample 2 in the rubbery state was higher, due to high-temperature crosslinking.

### 3.1. Effects of Temperature on the Elasticity Modulus

**Figure 2:**
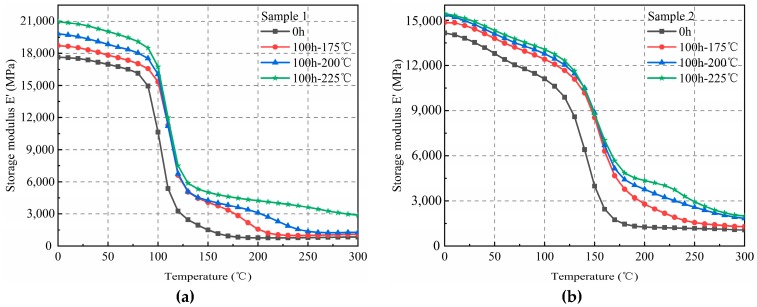
Elasticity modulus of EMC(Epoxy Molding Compound) after 100 hr of aging at three different temperatures. (**a**) Sample 1; (**b**) Sample 2.

After 100 h of thermal aging, the storage temperature and the numerical value of the elasticity modulus of sample 1 (left) increased continuously, and for the glassy and rubbery states, the increased value was close (Figure 2). Similarly, after 100 h of thermal aging, the elasticity modulus of sample 2 (right) increased continuously with an increase in temperature. The increasing trends in the glassy and rubbery states were similar to those found for sample 1.

**Figure 3:**
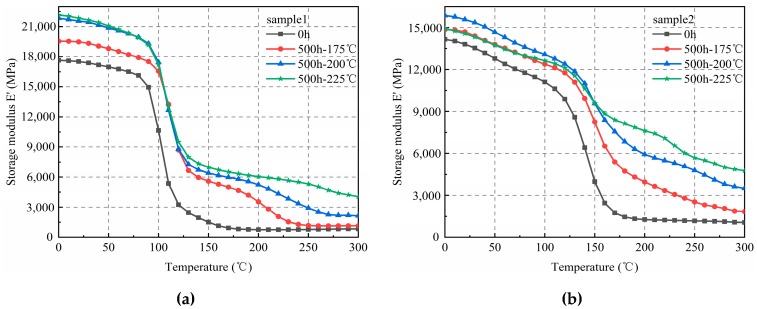
Elasticity modulus of EMC at 500 h of thermal aging at three different temperatures. (**a**) Sample 1; (**b**) Sample 2.

The variation curves of the elasticity modulus of samples 1 and 2 according to temperature, after 500 h of thermal aging, are shown in Figure 3. Similar trends as those found in Figure 2 were observed. Therefore, with the same aging time, the elasticity moduli of the samples correlated positively with the temperature. The increase in the elasticity modulus in the high-elasticity state was significantly higher than that in the glassy state.

### 3.2. Effects of Thermal Aging Time on the Elasticity Modulus

**Figure 4:**
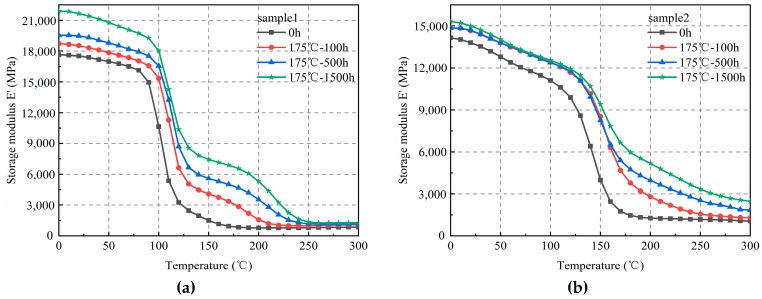
The elasticity modulus of EMC under different aging times (temperature = 175 °C). (**a**) Sample 1; (**b**) Sample 2.

At a fixed storage temperature of 175 °C, the numerical value of the elasticity modulus of sample 1 (left) increased with an increase in the aging time. A similar result was observed for sample 2 (right) (Figure 4).

**Figure 5:**
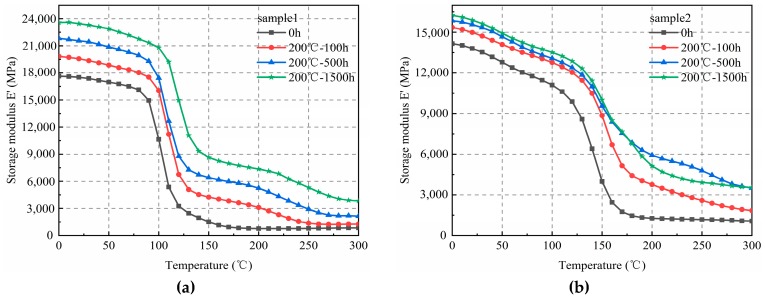
Elasticity modulus of the EMC at different aging times (temperature = 200 °C). (**a**) Sample 1; (**b**) Sample 2.

The curves showing the relationship between the elasticity modulus and the aging time of the two samples at 200 °C are shown in Figure 5. Similar trends as those discussed in Figure 4 were observed. Therefore, at the same aging temperature, the numerical value of the elasticity modulus correlated positively with aging time.

**Figure 6:**
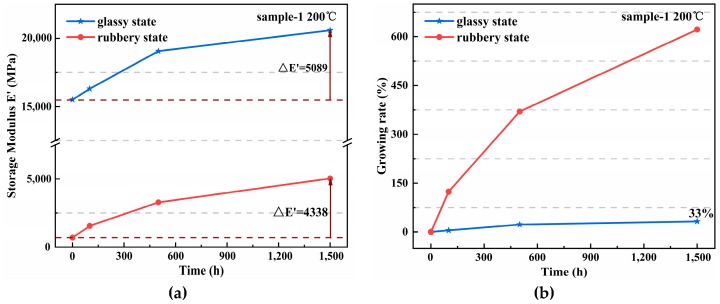
Increment and growing rate in the average elasticity modulus of sample 1 (temperature = 200 °C). (**a**) Increment; (**b**) Growing rate.

Moreover, the increment and the growing rate of the average elasticity modulus for sample 1 at 200 °C are shown in Figure 6. The increments of the glassy and rubbery states were similar, and the maximum increments were at 5089 and 4338 MPa, respectively. However, the growth rate was significantly different, and the growth of the rubbery state (618%) was significantly higher than that of the glassy state (33%). Similar trends to these were observed in sample 2.

### 3.3. Effects of Filling Contents on the Elasticity Modulus and Material Failure Analysis

**Figure 7:**
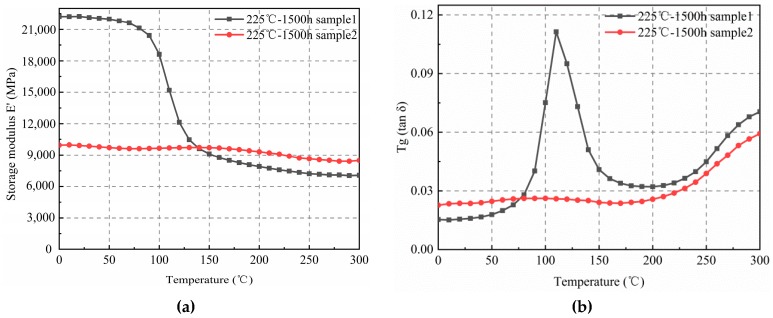
Elasticity modulus and *T*_g_ of samples after 1500 h of high-temperature aging at 225 °C. (**a**) E’–T curves; (**b**) tan δ–T curves.

Increasing the temperature (225 °C) and aging time (1500 h) resulted in the elasticity modulus of sample 1 increasing continuously in both the glassy and rubbery states (Figure 7a). However, the modulus of sample 2 decreased in the glassy state, while in the rubbery state, it increased. The curve tended to be linear. The *T*_g_ curve shown in Figure 7b also indicated that sample 2 had no peak at 165 °C, suggesting that sample 2 had been oxidized completely after long-term high-temperature storage and reduced the mechanical properties of polymer materials. This indicates that when the filler content was low, the corresponding epoxy resin content increased, and the EMC was oxidized more readily. In contrast, when the filler was silica powder, a high melting point and oxidation resistance resulted. Therefore, when the content was high, the proportion of silica in the space of the molding compound increased, inhibiting the oxidation process. The filler had a retarding effect on the thermal oxidation of EMC, and the content of the filler directly affected the degree of oxidation of the epoxy molding compound.

**Figure 8:**
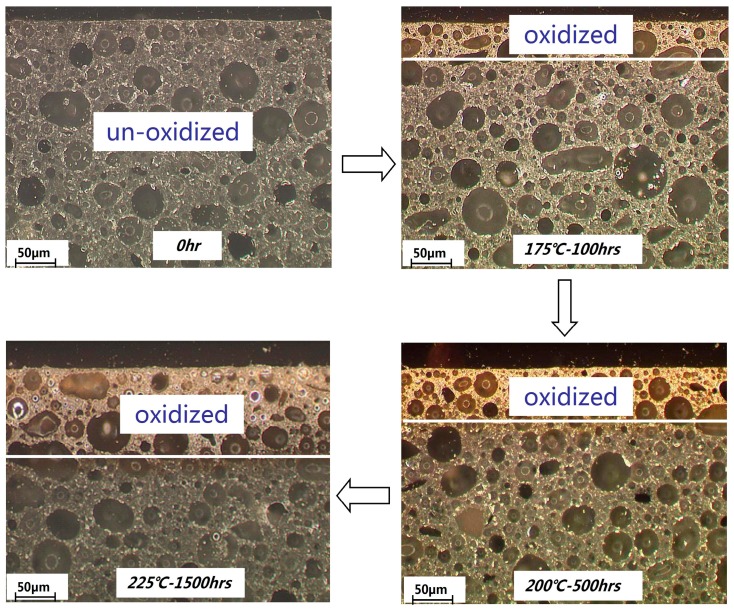
Microscopic images of sample 1 at different aging temperatures and times.

**Figure 9:**
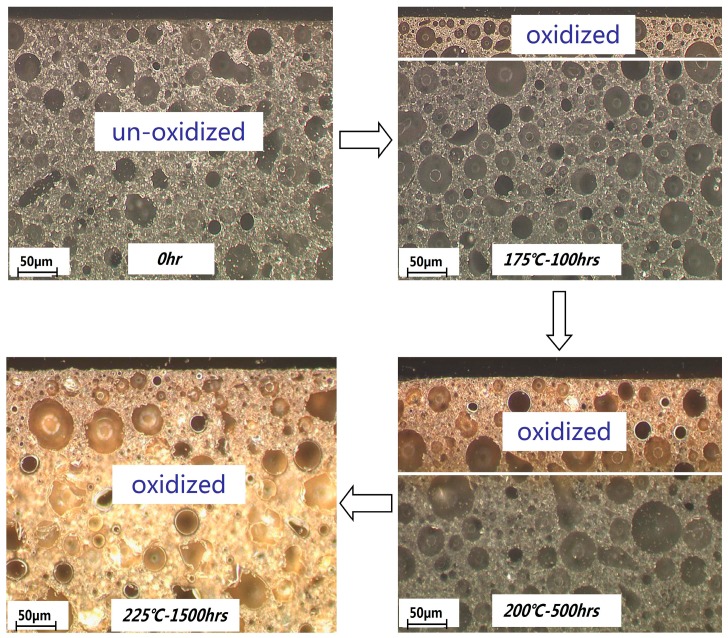
Microscopic images of sample 2 at different aging temperatures and times.

The microscopic images of samples 1 and 2 after thermal oxidation are shown in Figure 8 and Figure 9. As the molding compound aged, the colors of the materials under the microscope gradually changed from black to yellow. The difference in color depth of the photo is due to the difference in the filler content. It can be observed that as the aging temperature and time increased, the thickness of the oxide layer also increased. However, when the temperature reached 225 °C and the aging time exceeded 1500 h, the thickness of the oxidation layer in sample 1 with a high filling content was less than half the original value, while sample 2 (filling content) was mostly oxidized. This was in agreement with the curve analysis shown in Figure 7.

Temperature, aging time, and filling content were the key factors influencing the degree of thermal oxidation of EMC. In general, the elasticity moduli of the EMCs increased with increasing temperature and aging time. The temperature had a greater influence on the degree of thermal oxidation of EMC. When the aging temperature was increased above 250 °C, the EMC was completely oxidized, and its original characteristics disappeared. When the temperature was below 225 °C, the surface oxide layer formed by thermal aging for most materials reduced the further oxidation of the EMCs. A low filling content resulted in the material developing phase changes after long-term high-temperature oxidation, and reduced polymer mechanical properties. The elasticity modulus in the high-temperature zone was much higher, and the material became hard and brittle [12]. Therefore, cracking failures developed easily, thereby causing failure in the electronic elements. When the material was not completely oxidized, an interface that was not oxidized was formed between the oxide layer and the inner core. Due to the different mechanical properties of the materials, such as the elasticity modulus, the thermal expansion coefficient, Poisson’s ratio, etc., a large internal stress forms at the interface, which eventually leads to cracking and failure of the material [12,14].

## 4. Conclusions

The evolution of the elasticity modulus under the high-temperature aging of EMC using DMA technology was investigated. A qualitative analysis of the relationship between material failure and the elasticity modulus was performed, and the following conclusions were made:

(1) The filling content (silica powder) of EMC directly affected the elasticity modulus and *T*_g_ value of the materials. The elasticity modulus of silica was larger than that of the epoxy resin. Therefore, a higher filler content in EMC resulted in a greater elasticity modulus of the material. However, excessive filler affected the curing of the epoxy resin, and a lowering of the glassy transition temperature.

(2) With an increase in the thermal aging time and temperature, the elasticity modulus of the EMC increased continuously. The increments of the glassy and rubbery states were similar. However, the growing rate was significantly different, and the growth of the rubbery state was significantly higher than that of the glassy state.

(3) The degrees of oxidation of the materials under high temperatures correlated negatively with the filling content (silica powder) in the EMC. After long-term high-temperature oxidation, a phase change occurred, and the polymer mechanical properties were reduced. An interface formed between the oxide layer and the inner core (not oxidized) when the material was not completely oxidized. Due to the different mechanical properties of the materials, such as their elasticity moduli, thermal expansion coefficients, Poisson’s ratios, etc., a large internal stress formed at the interface, which eventually led to cracking and failure of the material [12,14].

## Figures and Tables

**Table 1:** The Experimental Conditions.

Filling Contents	Aging Temperatures	Aging Times
High (material 1) Low (material 2)	175 °C 200 °C 225 °C	0 h (Unaged) 100 h 500 h 1500 h

**Table 2:** Mechanical parameters of non-aged (0 h) samples. E^1^ is the mean modulus of the glassy state, and E^2^ is the mean modulus of the rubbery state.

Material	Filling Content	E^1^/MPa	E^2^/MPa	T_g_ (tan)/°C
Sample 1	High (89%)	16,723	815	109
Sample 2	Low (79%)	12,343	1235	152
